# Determination of nicotine in newborn meconium by high-Resolution ambient mass spectrometry using wooden-Tip spray

**DOI:** 10.3389/fchem.2023.1122137

**Published:** 2023-01-19

**Authors:** Xinrong Wang, Mingyu Yang, Hui Xiao, Danping Liu, Lu Pan, Liuyang Zhang, Yan Yang, Qing Lu, Yanqiu Liu, Xiao Yang, Bicheng Yang

**Affiliations:** ^1^ Jiangxi Key Laboratory of Birth Defect Prevention and Control, Jiangxi Maternal and Child Health Hospital, Nanchang, China; ^2^ Maternal and Child Health Hospital of Nanchang Medical College, Nanchang, China; ^3^ The First Clinical Medical School, Guangdong Medical University, Zhanjiang, China

**Keywords:** wooden tip, meconium, nicotine, ambient mass spectrometry, electrospray ionization

## Abstract

Prenatal exposure to nicotine that are mainly produced from tobacco smoke has been reported to affect infants. Therefore, nicotine exposure is one of important health concerns for newborn screening. Detecting nicotine and its metabolites such as cotinine in meconium were widely used to evaluate the tobacco exposure of pregnancy. In this study, disposable wooden tips were applied for touch sampling of meconium from newborn infants, and then were directly mounted on mass spectrometer (MS) to perform rapid screening of nicotine and cotinine. Choice of extraction/spray solvents was optimized. The limits of detection, reproducibility, linear response for direct analysis of meconium were also investigated. It is found the limits of detection (S/N = 3) to be as low as 0.36 ng/mg and 1.18 ng/mg for nicotine and cotinine, respectively, while the limits of quantitation (S/N = 10) to be 1.19 ng/mg and 3.94 ng/mg for nicotine and cotinine, respectively. The relative standard deviations (RSD) were found to be at 8.4%–19.8% (n = 6) for nicotine and cotinine, a good linear range from 5–500 ng/mL (*R* ^2^ > 0.99). These analytical performances are well-accepted levels for ambient mass spectrometer analysis. In this study, evaluation of nicotine and cotinine in 22 puerpera volunteers were conducted by the established wooden-tip spray mass spectrometry (WTS-MS). These results showed that wooden-tip spray mass spectrometry would be useful for newborn screening of nicotine and cotinine in meconium with high reproducibility, speed, sensitivity, and specificity. Owing to the use of disposable wooden tips that involves no sample preparation and no chromatographic separation, our results show that wooden-tip spray mass spectrometry is a powerful tool for determination of nicotine in newborn meconium.

## Introduction

Nicotine is one of the most popular air pollutants (Bruin et al., 2010; Gibbs et al., 2016). There remains a potential reproductive health risk during passive nicotine consumption, which is mainly generated from tobacco smoke or electronic nicotine (Wong et al., 2015; Cardenas et al., 2019; Price and Martinez, 2019). For example, pregnant tobacco exposure is associated with many health risks, including premature rupture of membranes, placenta previa, placental abruption, preterm delivery, shortened gestation, fetal growth restriction and low birth weight, sudden infant death syndrome, perinatal mortality, as well as cognitive and neuro developmental disorders (Slotkin et al., 1995; Wickstrom, 2007). Nicotine, the special maker of tobacco, can be used an indicator to directly reflect the impact of tobacco smoke; while the cotinine, a metabolite of nicotine, can be used for evaluating the effect of nicotine exposure and metabolism. There are many clinical samples used for evaluation of tobacco smoke by detecting nicotine and cotinine. Tissue, blood, and urine are usually used for evaluating the nicotine level (Urakawa et al., 1994; Hoofnagle et al., 2006). Among these clinical samples, meconium is useful sources to evaluate the newborn exposure. Meconium is accumulated from 12th–13th weeks of gestation in intestinal compartments to birth, and has been well accepted to be a useful clinical sample (Ostrea Jr et al., 1994; Baranowski et al., 1998; Chan et al., 2004; Gray et al., 2010; Himes et al., 2013; Meyer-Monath et al., 2014; Naritaka et al., 2015). The meconium is usually expelled by the newborn within 24 h after birth. Unlike other fetal biofluids and biomaterials such as urine, blood and hair, the collection of viscous meconium is easy-operation and non-invasiveness for the newborns. Therefore, rapid screening the nicotine and cotinine would be useful for evaluating the nicotine exposure of fetus during pregnancy in tobacco environments.

Mass spectrometry is a powerful analytical platform for disease diagnosis and research, and has been developed for biological and clinical analysis (Fung et al., 2020; Hu and Ouyang, 2021; Yuan and Hu, 2021; Hu, 2022). To determine nicotine in meconium, liquid chromatography-mass spectrometry (LC-MS) and gas chromatography-mass spectrometry (GC-MS) are usually used (Baranowski et al., 1998; Chetiyanukornkul et al., 2004; Yuan et al., 2013; Yuan et al., 2018). Because the meconium is a viscous complex sample, complicated sample preparations and chromatographic separations are required before MS analysis. Current point-of-care (POC) testing requires simple, rapid, sensitive analytical method to perform the fast identification and quantification of target analytes for evaluating follow-up diagnosis and medical management (Ferreira et al., 2016). Therefore, the new application of simple, rapid and reliable analytical tools are highly needed for newborn screening (Yang et al., 2016).

Recently, ambient MS with ambient ionization techniques have been applied for detecting trace analytes in complex clinical samples with no or little sample pretreatment (Ferreira et al., 2016). For example, desorption electrospray ionization, direct analysis in real time, and other ambient desorption/ionization techniques have been successfully applied clinical analysis. Moreover, Similarly, direct electrospray ionization (ESI) techniques have also developed for direct extraction/ionization under ambient conditions (Ferreira et al., 2016). Moreover, various solid substrates such as metal foil (Hu et al., 2014), porous paper (Cai et al., 2021), and biological tissue (Hu et al., 2012), were used for direct loading raw samples, and then were performed ESI emitters (Klampfl and Himmelsbach, 2015). Among these ambient ESI techniques, ESI on wooden tip is one of powerful ambient ESI techniques (Hu et al., 2011; Hu and Yao, 2018; Ng et al., 2019; Millán-Santiago et al., 2022), provides a rapid method for rapid sampling and direct analysis of clinical samples (Hu and Yao, 2022). By using wooden tip, wooden tip was directly used for loading raw samples, and organic solvent was then loaded on the sample to extract analytes and to generate spray ionization by applying a high voltage on wooden tip. Under such conditions, a wooden-tip spray (WTS) was directly generated. WTS-MS have been proven a useful analytical tool in clinical analysis, provides a new analytical strategy for clinical analysis (Hu et al., 2011). Therefore, sample transfer and sample pre-treatment are avoided during WTS-MS analysis, providing a rapid method for direct sample analysis for clinical applications at point-of-care.

In this study, we demonstrated the rapid screening of nicotine and cotinine in meconium by using WTS-MS. The wooden tips were directly used for sampling meconium samples from infants. And then, the samples were directly extracted and ionized by optimized organic solvents. The blank meconium samples were spiked with nicotine and cotinine to investigate the analytical performances of WTS-MS. The sensitivity, reproducibility, and quantitation were investigated in this work. Overall, our results show that WTS-MS is promising method for rapid screening of nicotine and cotinine in meconium.

## Materials and methods

### Chemicals and materials

Wooden tips (common toothpicks) were purchased from the local supermarket in Nanchang. The toothpicks are labeled to be made of natural wood without chemical modification. Before use, wooden toothpicks were washed by methanol and water to clean the surface contaminants. Nicotine and cotinine standards were purchased from Dr Ehrenstorfer Gmbh (Augsburg, Germany). Isotopic internal standards (IS), nicotine-d4 and cotinine-d3, were purchased from Gerilliant (Round Rock, TX, United States). Pure HPLC-grade organic solvents such as hexane, acetonitrile, methanol, and ethanol were bought from the Chinese Chemical Reagent Co., Ltd. (Shanghai, China). All water used in this study is Milli-Q water.

### Collection of clinical meconium samples

All clinical meconium samples were from 22 puerpera volunteers from Jiangxi Provincial Neonatal Screening Center (Nanchang, China). Meconium samples were collected over the first 3 days of life and were immediately stored at −80 C before use. All puerperae have signed the informed consent forms for this study. This study was approved by the Ethics Committee of Jiangxi Provincial Maternal and Child Health Hospital (Nanchang, China).

### Wooden-tip spray mass spectrometry analysis

**FIGURE 1:**
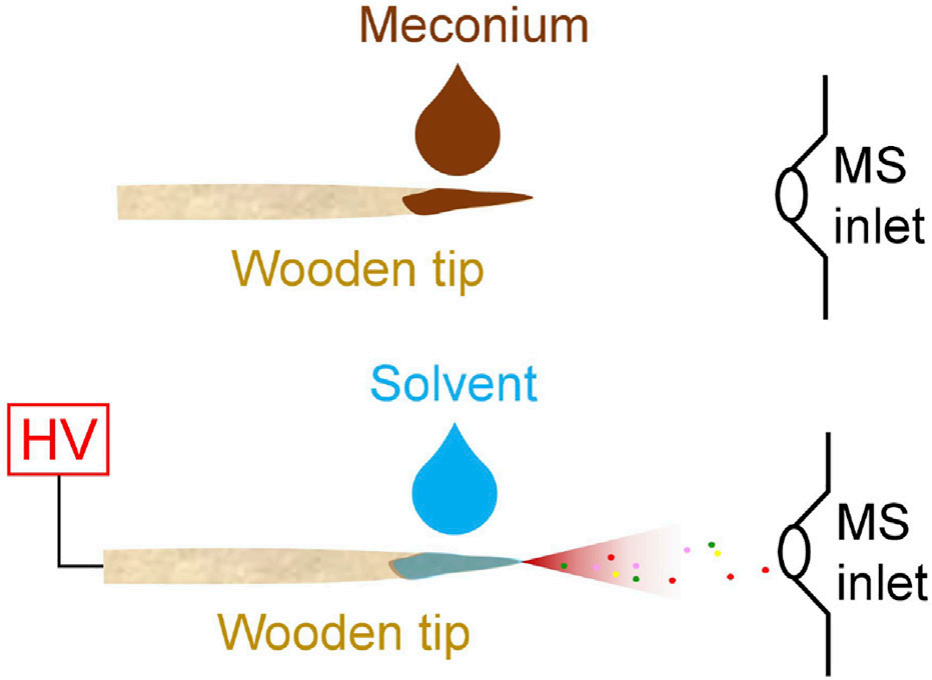
WTS-MS analysis of meconium samples: sample loading (upper), direct spray ionization (lower).

As shown in Figure 1, a pre-cut wooden tip (tip-end: ∼0.2 mm, diameter: 2.0 mm, length: 1.5 cm) was fixed at a nano-ESI device (Thermo Fisher Scientific, Bremen, Germany). Viscous meconium sample (1 µL) was directly loaded on wooden tip (Figure 1A) by pipette, and then 5.0 µL of solvent was loaded on sample surface that loaded on wooden tip and connected a high voltage (Figure 1B). Particularly, under the optimized solvent, methanol solution containing internal standard, nicotine-d4 (10 ng/mL) and cotinine-d3 (10 ng/mL), was slowly added to sample *via* pipette tip to prepare a fixed amount. Under such conditions, the wooden tip that contained internal standards and potential analytes (nicotine and cotinine) was fixed in front of the MS inlet. The distance between wooden tip-end and MS inlet is ∼10.0 mm. High voltage (+3.5 kV) was applied to the wooden tips, and the spray ionization could be generated. All experiments were carried on the mass spectrometer (Thermo Fisher Scientific, Bremen, Germany), which could perform tandem mass spectrometry experiments. All the mass spectral data acquisition and instrumental control were conducted by using Xcalibur 3.0 software. The acquisition speed was 20 scans/sec. Typically, first 0.5 min of signal duration was averaged to obtain the high-resolution mass spectra. Under MS/MS experiments, the isolation window is 0.4 Da and the collision energy is 25% for nicotine and cotinine.

## Results and discussion

### Detection and identification of nicotine and cotinine

**FIGURE 2:**
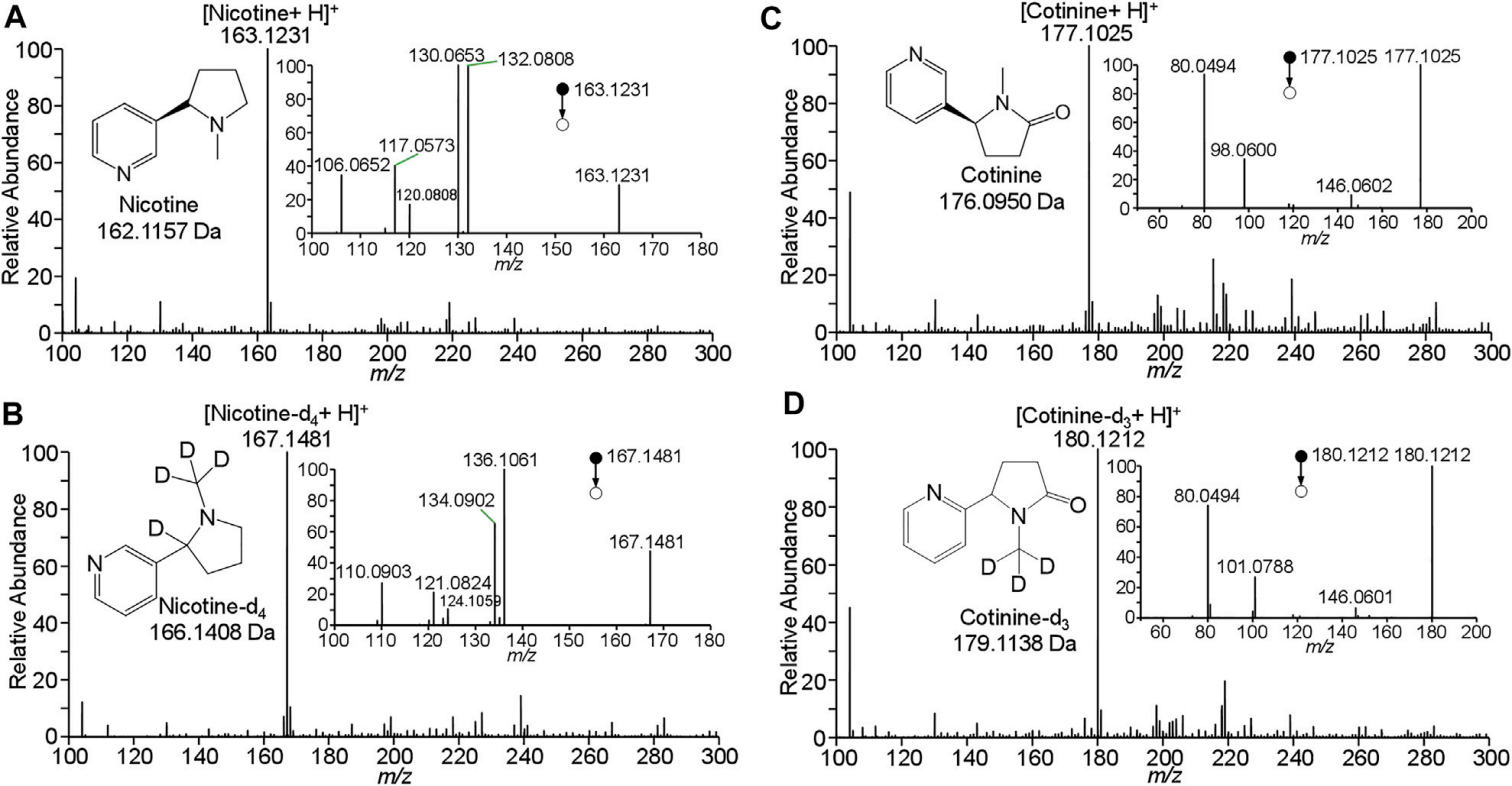
WTS-MS detection of nicotine and cotinine: **(A)** nicotine, **(B)** nicotine-d4, **(C)** cotinine, **(D)** cotinine-d3.

To determinate nicotine and cotinine, their standard solutions were analyzed by WTS-MS to obtain their characteristic mass spectra. Figure 2A shows WTS-MS detection of nicotine standard at 10 ng/mL (5.0 μL). The based peak at m/z 163.1231 clearly shows the detection of protonated nicotine (M + H)^+^ (calculated m/z 163.1235) with high signal-to-noise (S/N), showing an accurate high-resolution mass spectrum with an ultra-low mass error at 2.5 ppm. Although there are some background signals at a low relative abundance, the trace nicotine on wooden tip is ambiguously detected, due the highly efficient ionization of WTS. Upon MS/MS, the characteristic fragment ions at m/z 106.0652, m/z 117.0573, m/z 120.0808, m/z 130.0653, and 163.0808 were observed, as shown in inset of Figure 2A. Because nicotine is a bicyclic compound with a pyridine cycle and a pyrrolidine cycle, these fragment ions were produced by the dissociation of pronated nicotine. To further identify the nicotine, detection of isotopic nicotine-d4 was also performed by WTS-MS/MS, as shown in Figure 2B. The based peak at m/z 167.1481 is corresponding to the protonated nicotine-d4 (M + H)^+^. Upon MS/MS, the characteristic fragment ions at m/z 110.0903, m/z 121.0824, m/z 124.1059, m/z 134.0902, and 136.106 were observed (inset of Figure 2B). It is found that these fragment ions are corresponding to residues of n-methylpyrrolidine in the nicotine molecule, because the H/D sites are remined in these fragment ions (Figure 2B).

Figure 2C shows WTS-MS detection of cotinine standard at 10 ng/mL (5.0 μL). The based peak at m/z 177.1025 display the protonated cotinine (M + H)^+^ with high signal-to-noise (S/N). Upon MS/MS, the characteristic fragment ions at m/z 80.0494, m/z 98.0600, and m/z 146.0602 were observed (inset of Figure 2C). Because cotinine also contains a pyridine cycle and a pyrrolidine cycle, these fragment ions were produced by the dissociation of pronated cotinine. The fragment ions at m/z 146.0602 is corresponding to the loss CH_3_-NH_2_ (calculated 31.0422 Da) from protonated cotinine. The ions at m/z 80.0494 is corresponding to the protonated pyridine (calculated m/z 80.0500), while the ions at m/z 98.0600 is the fragment ions of by loss of pyridine (calculated 79.0422 Da) from protonated cotinine. These fragment ions were further confirmed by the isotopic cotinine-d3, as shown in Figure 2D. There is base peak of pronated cotinine-d3 (M + H)^+^at m/z 180.1212. Because three H/D sites are in the pyrrolidine cycle. The ions at m/z 146.0602 is produced by the loss of CD_3_-NH_2_ from protonated cotinine. The peak at m/z 80.0494 is confirmed for protonated pyridine (calculated m/z 80.0500), while the ions at m/z 101.0788 is produced from protonated cotinine-d3 by loss of pyridine, as shown inset of Figure 2C. These data clearly show the detection and identification of nicotine and cotinine by high-resolution and MS/MS spectra. The results of characteristic mass spectra are useful for further identification and quantification of nicotine and cotinine in complex clinical samples.

### Quantitative detection of nicotine and cotinine in meconium

**FIGURE 3:**
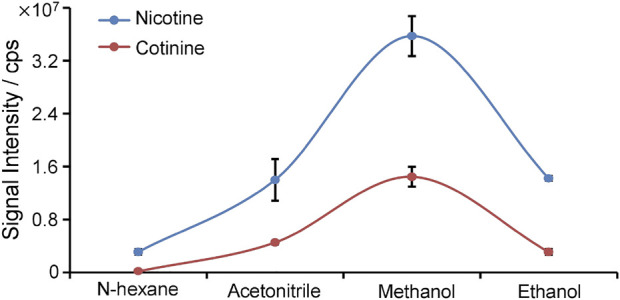
Spray and extraction of solvent of WTS-MS.

**FIGURE 4:**
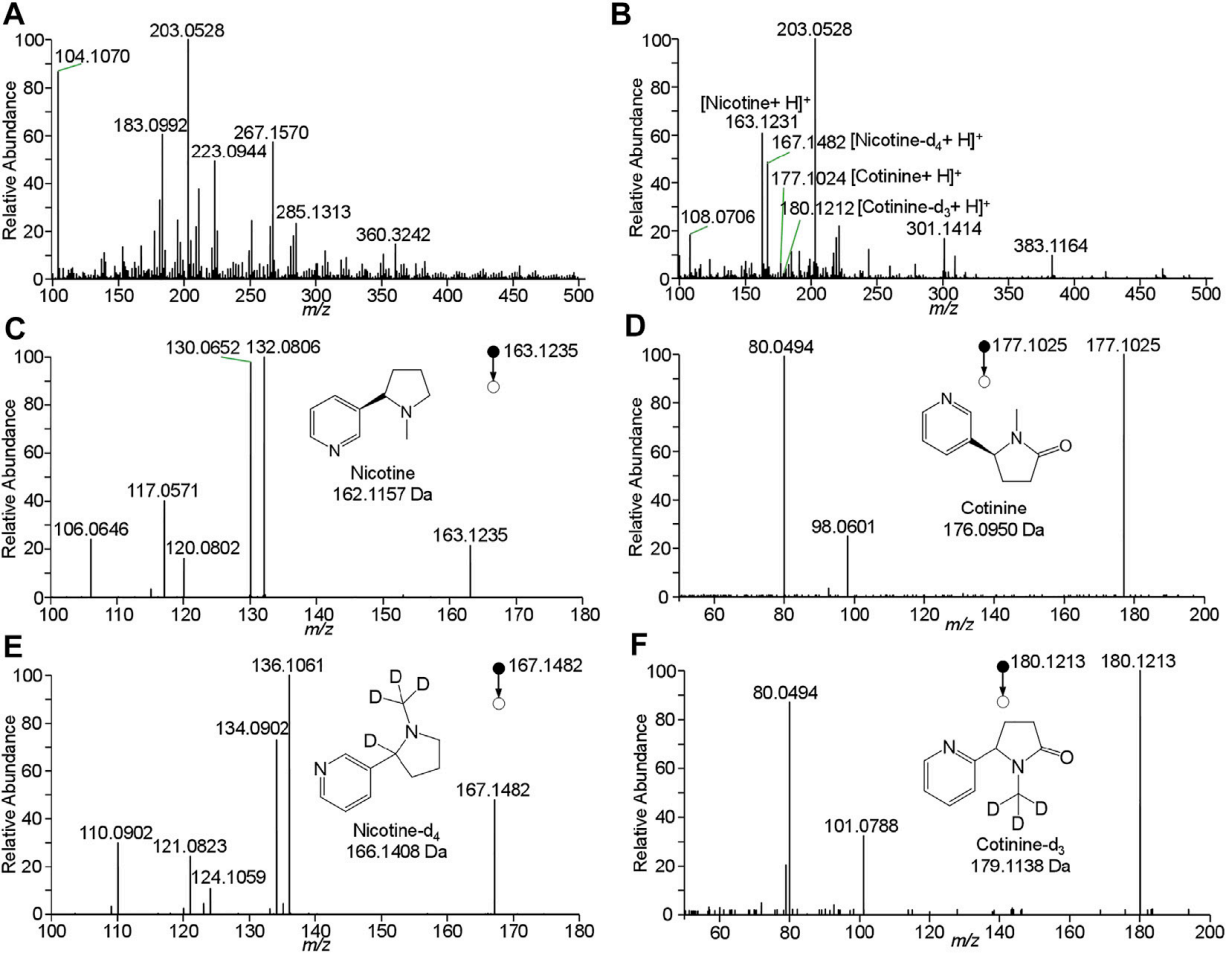
WTS-MS detection of nicotine and cotinine in meconium: **(A)** blank meconium, **(B)** meconium spiked with nicotine and cotinine, **(C)** MS/MS spectrum of nicotine, **(D)** MS/MS spectrum of cotinine, **(E)** MS/MS spectrum of nicotine-d4, **(F)** MS/MS spectrum of cotinine-d3.

To improve the detection of nicotine and cotinine, the spray solvent was optimized to extract and ionize nicotine and cotinine. Pure nicotine and cotinine solution was loaded on wooden tip and was dried in the air, and the solvent was loaded onto wooden tip for extracting the analytes and generating spray ionization. In this study, various organic solvents including n-hexane, acetonitrile, methanol, and ethanol, which are common organic solvent and can used for wooden-tip ESI under ambient conditions (Hu and Yao, 2018; Huang et al., 2019), were investigated by WTS-MS, as shown in Figure 3. It is found that the highest signal responses of nicotine and cotinine were obtained by use of methanol. Under the optimized solvent (i.e., methanol), meconium samples were analyzed by WTS-MS. As shown in Figure 4A, the high-resolution mass spectrum of blank meconium samples was obtained, showing various metabolites in meconium samples. However, in this negative meconium sample, no nicotine and cotinine were found in the high-resolution mass spectrum and MS/MS spectra; Figure 4B shows WTS-MS spectrum obtained from meconium samples spiked with nicotine (50 ng/mL), cotinine (50 ng/mL) and their isotopic internal standards (nicotine-d4: 10.0 ng/mL, cotinine-d3: 10. ng/mL). In the high-resolution spectrum, although there are many peaks of biomatrices of meconium, the protonated analytes, including nicotine, cotinine, nicotine-4, and cotinine-3, are also observed according to their special mass-to-charges. Upon MS/MS experiments, these target analytes were further confirmed, as shown in Figures 4C–F, which are good agree with the MS/MS spectra obtained from standards (Figure 2). By using WTS-MS, sampling and analysis of single sample can be completed with minutes, showing a rapid screening. The results show that WTS-MS is promising method for detecting trac nicotine and cotinine in meconium samples.

To further investigate the quantitative analysis of nicotine and cotinine in meconium samples, the analytical performances of WTS-MS were investigated. To compensate the variations of WTS-MS, the limits of detection (LOD) and limits of quantitation (LOQ) were determined by comparing the ratios of signal intensity of spiked samples (i.e., nicotine and cotinine) and blank sample with internal standards (i.e., nicotine-d4 and cotinine-d3) and thus the ratios of (I_analyte_/I_IS_)_spiked_/(I_analyte_/I_IS_)_blank_ were obtained, as shown in Table 1. The LOD (S/N = 3) and LOQ (S/N = 10) of nicotine were found to be 0.36 ng/mL and 1.19 ng/mL, respectively, while LOD (S/N = 3) and LOQ (S/N = 10) of cotinine were found to be 0.18 ng/mL and 3.94 ng/mL, respectively. These data indicated that WTS-MS is a sensitive method for detecting trace nicotine and cotinine in complex meconium. To investigate the reproducibility of WTS-MS, different concentrations of nicotine and cotinine were analyzed, as shown in Table 2. It found that relative standard deviations (RSDs) were found to be at 9.8%–12.6% (n = 6) for six repeated detections of nicotine at low (5.0 ng/mL), middle (50.0 ng/mL), and high concentration (500.0 ng/mL), respectively. At same concentrations, RSDs of cotinine were found to be 8.4%–11.0% (n = 6). These data show the WTS-MS is reliable method of ambient MS analysis of meconium samples.

**TABLE 1:** LODs and LOQs of nicotine and cotinine.

Analytes	LOD (S/N = 3)	LOQ (S/N = 3)
Nicotine	0.36	1.19
Cotinine	1.18	3.94

**TABLE 2:** RSDs (n = 6) of nicotine and cotinine.

Analytes	Low concentration RSD (n = 6)	Middle concentration RSD (n = 6)	High concentration RSD (n = 6)
Nicotine	9.8	19.8	12.6
Cotinine	8.4	11.0	9.5

**FIGURE 5:**
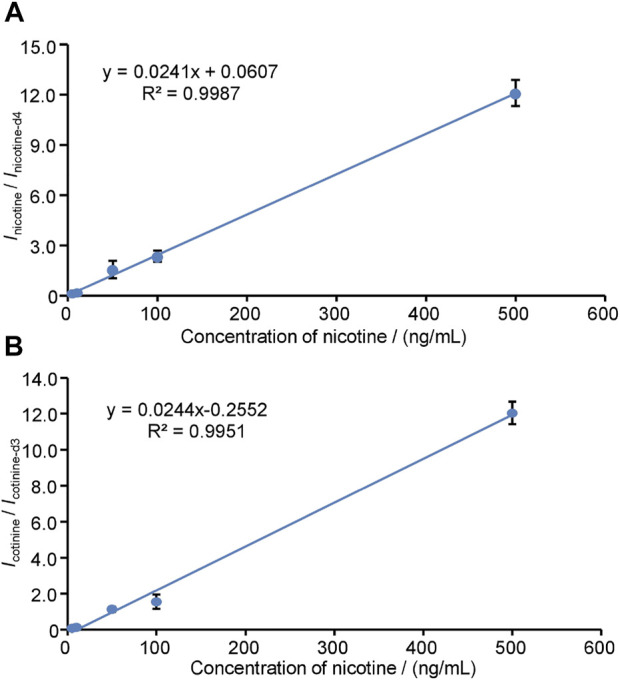
Linear responses obtained by WTS-MS using internal standards: **(A)** nicotine, **(B)** cotinine.

To conduct the quantitative detection of nicotine and cotinine, the linear responses of nicotine (Figure 5A) and cotinine (Figure 5B) were obtained ranging from 5.0 ng/mL to 500.0 ng/mL using fixed internal standards (nicotine-d4 for nicotine, cotinine-d3 for cotinine). The calibration was constructed by averaging five sets of experimental data of the ratios of nicotine-to-nicotine-d4 and cotinine-to-cotinine-d3. The use of internal standards provides excellent performances to compensate fluctuation of WTS-MS processes, showing a wide linear range and good coefficients (*R* ^2^ > 0.99). In this study, further evaluation of nicotine and cotinine in 22 puerpera volunteers were conducted by WTS-MS, the results show that nicotine and cotinine are below the LODs, showing there is ultra-low health risk of nicotine exposure. Owing to advantages of disposable wooden tips that involves no sample preparation and no chromatographic separation, our results show showed that WTS-MS is useful tool for newborn screening of nicotine and cotinine in meconium with high reproducibility, speed, sensitivity, and specificity.

## Conclusion

In this work, WTS-MS was used for detection of nicotine and cotinine in meconium samples. Compared to conventional MS method (Baranowski et al., 1998; Chetiyanukornkul et al., 2004; Yuan et al., 2013; Yuan et al., 2018), the advantage of WTS-MS for direct meconium analysis is that the disposable, simple, and low-cost wooden tip were used for sample loading and direct ionization without sample pretreatment and separation. In this work, trace nicotine and cotinine were successfully detected by high-resolution mass spectra and MS/MS spectra. Although no positive samples were observed, the sensitivity, specificity, reproducibility, and quantitation of WTS-MS were investigated by detection of spiked samples, showing good analytical performances for ambient MS analysis. Overall, our results show that WTS-MS is promising simple, rapid, and effective analytical tool for detection of nicotine and continue in newborn screening. Due the complicated matrices in clinical samples, further improvement for sensitive and accurate detection of trace analytes is required, the fast microextraction techniques such as solid-phase microextraction is high expected in the future (So et al., 2019; Hu and Ouyang, 2021).

## Data Availability

The original contributions presented in the study are included in the article/supplementary material, further inquiries can be directed to the corresponding authors.
